# Geographic Divergence of Bovine and Human Shiga Toxin–Producing *Escherichia coli* O157:H7 Genotypes, New Zealand^1^

**DOI:** 10.3201/eid2012.140281

**Published:** 2014-12

**Authors:** Patricia Jaros, Adrian L. Cookson, Donald M. Campbell, Gail E. Duncan, Deborah Prattley, Philip Carter, Thomas E. Besser, Smriti Shringi, Steve Hathaway, Jonathan C. Marshall, Nigel P. French

**Affiliations:** Massey University, Palmerston North, New Zealand (P. Jaros, D. Prattley, J.C. Marshall, N.P. French);; AgResearch Ltd, Palmerston North (A.L. Cookson);; Ministry for Primary Industries, Wellington, New Zealand (D.M. Campbell, G.E. Duncan, S. Hathaway);; Institute of Environmental Science and Research Ltd, Porirua, New Zealand (P. Carter);; Washington State University, Pullman, Washington, USA (T.E. Besser, S. Shringi)

**Keywords:** molecular epidemiology, Shiga toxin–producing *Escherichia coli* O157:H7, genotype, Shiga toxin–encoding bacteriophage insertion typing, cattle, transmission, New Zealand, geographic divergence, population structure, proportional similarity index, enteric infections, bacteria

## Abstract

Shiga toxin-producing *Escherichia coli* (STEC) O157:H7 is a zoonotic pathogen of public health concern worldwide. To compare the local and large-scale geographic distributions of genotypes of STEC O157:H7 isolates obtained from various bovine and human sources during 2008–2011, we used pulsed-field gel electrophoresis and Shiga toxin–encoding bacteriophage insertion (SBI) typing. Using multivariate methods, we compared isolates from the North and South Islands of New Zealand with isolates from Australia and the United States. The STEC O157:H7 population structure differed substantially between the 2 islands and showed evidence of finer scale spatial structuring, which is consistent with highly localized transmission rather than disseminated foodborne outbreaks. The distribution of SBI types differed markedly among isolates from New Zealand, Australia, and the United States. Our findings also provide evidence for the historic introduction into New Zealand of a subset of globally circulating STEC O157:H7 strains that have continued to evolve and be transmitted locally between cattle and humans.

## Abstract 2

A historically introduced subset of globally circulating strains continue to evolve and be transmitted between cattle and humans.

Shiga toxin–producing *Escherichia coli* (STEC) O157:H7 and related non-O157 STEC strains are zoonotic pathogens that can cause severe gastrointestinal illness in humans; clinical signs and symptoms of disease range from diarrhea and hemorrhagic colitis to life-threatening hemolytic uremic syndrome (*1*,*2*). Ruminants, asymptomatic carriers of STEC, shed the pathogen in their feces, and are considered a primary source of foodborne and environmental outbreaks of STEC infection in humans (*3*).

The incidence of STEC infections in New Zealand has been among the highest in the world. In 2012, a total of 147 clinical STEC cases (3.3 cases/100,000 population) were notified, of which 142 were confirmed (*4*). Consistent with observations in previous years, the predominant serotype among the confirmed cases was O157:H7 (83.8%; 119/142). STEC became a notifiable disease in New Zealand in 1997, and since then, the annual number of notifications has increased steadily (*4*). Although the spatial distribution of STEC cases in New Zealand suggests an association with farming and other rural activities, limited epidemiologic data are available on the transmission pathways of STEC from cattle to humans.

The objectives of this research were to 1) compare the population structure and geographic distribution of different genotypes of STEC O157:H7 isolates from bovine and human sources in New Zealand; 2) assess evidence for localized transmission of STEC from cattle to humans in New Zealand; and 3) compare the genotype distribution of isolates from New Zealand with those from Australia, the predominant historic source of imported New Zealand cattle (*5*), and the United States. To investigate the molecular divergence of isolates, we used 2 molecular typing methods: Shiga toxin–encoding bacteriophage insertion (SBI) typing and pulsed-field gel electrophoresis (PFGE) profiling. Although PFGE can provide an indication of genomic similarities, it cannot provide a reliable measure of genetic relatedness of isolates, and the visual assessment of bands on an agarose gel to create PFGE profiles can result in misclassification bias (*6*). By using 2 methods and by examining the concordance between them, we could use the combined genotyping datasets to assess structuring and patterns of diversity among STEC O157:H7 isolates of bovine and human origin in New Zealand.

## Methods

### Human Isolates and Data

For the study, we obtained a total of 363 human-derived STEC O157:H7 isolates from the national Enteric Reference Laboratory (Institute of Environmental Science and Research Ltd, Upper Hutt, New Zealand) along with the associated PFGE profiles (restriction enzyme *Xba*I) and geographic data (North or South Island, New Zealand, and region on each island). Of the 363 isolates, 278 (76.6%) originated from the North Island. The isolates were from patients with clinical STEC infections that occurred in New Zealand during 2008–2011 and represent 71.3% (363/509) of the STEC O157 cases notified and confirmed during 2008–2011 (*7*). The cases were reported as sporadic cases or household clusters (i.e., 2 STEC infections in the same home) and were not associated with confirmed foodborne outbreaks.

### Bovine Fecal Isolates and Data

Fecal STEC O157:H7 isolates (n = 40) used in the study had been collected from cattle in previous studies conducted at beef slaughter plants in New Zealand during 2008 (*8*) and 2009–2011 (*9*). Data regarding the origin (North or South Island, region, farm location) of the cattle and the virulence profiles of the isolates (virulence genes *ehx*A, *eae*, *stx*1, *stx*2, and subtype *stx*2c) were available. The isolates were retrieved from feces samples collected from 26 calves and 14 adult cattle, most (80.0%, 32/40) of which were from the North Island; the animals originated from 35 farms.

### Bovine Meat Isolates and Data

Bovine meat isolates (n = 235) used in the study were from test samples used in routine mandatory testing at beef-processing plants across New Zealand during 2008–2011. Only PFGE profiles (*Xba*I) of STEC O157:H7 isolates were available for this study; the profiles were obtained from the national Enteric Reference Laboratory. Geographic data associated with meat-sample location (regions in North and South Islands) were obtained from the Ministry for Primary Industries (Wellington, New Zealand). Most isolates (85.5%, 201/235) originated from beef-slaughtering plants in the North Island. Virulence profiles of the isolates were not available.

### PCRs for Detection of Virulence Genes

All human isolates were regrown on Columbia Horse Blood Agar (Fort Richard Laboratories, Auckland, New Zealand). Bacterial DNA was extracted from 5 colonies by using 2% Chelex beads solution (Chelex 100 Resin; Bio-Rad, Richmond, CA, USA) and analyzed in 2 PCR assays by using an automated real-time thermocycler (Rotor Gene 6200HRM; Corbett Research, Mortlake, NSW, Australia).

A multiplex PCR assay was performed using previously published primer sequences to detect the presence of virulence genes encoding for enterohemolysin (*ehx*A) (*10*), intimin (*eae*) (*10*), and Shiga toxins (*stx*1 and *stx*2) (*11*). Primers for detection of genes *stx*1 and *stx*2 did not differentiate between subtypes of toxins. The final 25-μL PCR reaction volume contained 2× PCR buffer (Express qPCR SuperMix; Invitrogen, Carlsbad, CA, USA), 2 μmol/L of each primer, 2.0 μL of DNA, and 2.5 μL of sterile water. The amplification program included an initial enzyme-activation step of 5 min at 94°C, which was followed by 40 cycles of, 20 s at 94°C, 20 s at 64°C, and 20 s at 72°C, followed in turn by a final extension of 5 min at 72°C. The PCR products were detected by electrophoresis using a 2% (wt/vol) agarose gel (Agarose low EEO; AppliChem, Darmstadt, Germany) and then stained with ethidium bromide and visualized under ultraviolet illumination.

*stx*2-positive isolates were further tested to determine whether the *stx*2 gene that was present was the genetic subtype *stx*2c. The *stx*2c gene was detected by using previously published primer sequences (*12*,*13*). The final 20-μL PCR reaction volume contained 2× PCR buffer (Express qPCR SuperMix; Invitrogen), 2 μmol/L of each primer, 2.0 μL of DNA, and 6.0 μL of sterile water. The PCR included an initial enzyme-activation step of 5 min at 94°C, followed by 35 cycles of 20 s at 94°C and 20 s at 55°C; no extensions were used. The amplified PCR product was detected as described above.

### Molecular Typing Methods

All human and bovine fecal isolates were genotyped by using SBI typing (*12*,*13*); SBI typing is a multiplex PCR method for screening specific *stx*-associated bacteriophage insertion sites and *stx* genes (*stx*1 and genetic subtypes *stx*2a and *stx*2c of *stx*2). The characters A, W, Y, S and 1, 2a, 2c represent bacteriophage insertion sites *argW*, *wrbA*, *yehV*, *sbcB*, and Shiga toxin genes *stx*1, *stx*2a, *stx*2c (2 subtypes of *stx*2), respectively (*12*,*14*). All bovine fecal isolates were subtyped by using PFGE (*Xba*I) according to the standardized laboratory protocol published by PulseNet International (*15*). The SBI typing was completed at Washington State University, Pullman, Washington, USA.

### Location of Work and Ethical Approval

This work was completed at the Molecular Epidemiology and Public Health Laboratory, Infectious Disease Research Centre, Hopkirk Research Institute, Massey University, Palmerston North, New Zealand. The use of STEC isolates from clinical case-patients in New Zealand was approved by the Multi-region Ethics Committee, Wellington, New Zealand, on March 19, 2012; reference number MEC/11/04/043.

### Data Management and Statistical Analysis

For initial analysis, SBI types were grouped into 4 categories of 3 predominant SBI types (AY2a, WY12a, and ASY2c/SY2c) and other, less common, SBI types (AS12c, AS2c, ASWY2c, ASY12c, ASY2a2c, AWY12a, AWY2a, SWY2c, and Y2c). SBI types SY2c and ASY2c were grouped together because both were relatively common and shared the same virulence gene profile.

Although bovine meat isolates were not SBI-typed, a close correlation between PFGE profile and SBI type was observed for the human samples (Technical Appendix Figure 1) and the bovine fecal samples (Technical Appendix Figure 2). On the basis of the PFGE/SBI clusters, the most likely SBI type was inferred from the PFGE profiles for the meat isolates by taking the following approach. First, BioNumerics software version 6.6 (Applied Maths, Sint-Martens-Latem, Belgium) was used to compare PFGE profiles of human and bovine fecal isolates by conducting an UPGMA (unweighted pair group method with arithmetic mean) cluster analysis using the Dice similarity coefficient, with a band matching tolerance of 1%. Second, the UPGMA cluster analysis was applied on PFGE profiles of bovine meat isolates. The dominant SBI types in human and bovine fecal isolates were used to assign SBI-like types (AY2a, WY12a, and ASY2c/SY2c) to clusters with similar PFGE band patterns in bovine meat isolates.

**Figure 1:**
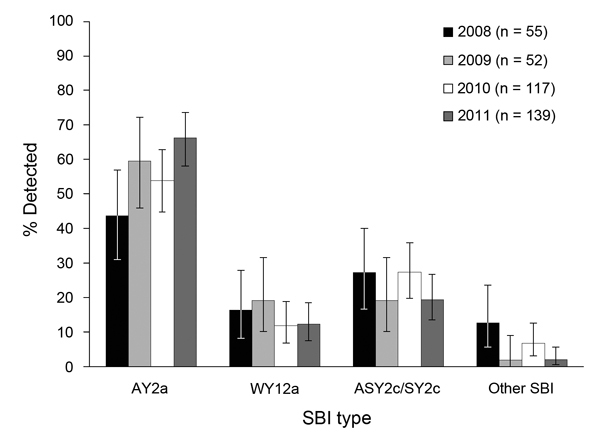
Proportional distributions, stratified by year, of Shiga toxin–encoding bacteriophage insertion (SBI) types AY2a, WY12a, and ASY2c/SY2c of 363 human Shiga toxin–producing *Escherichia coli* O157:H7 isolates from clinical case-patients in New Zealand, 2008–2011. Error bars indicate 95% CIs.

**Figure 2:**
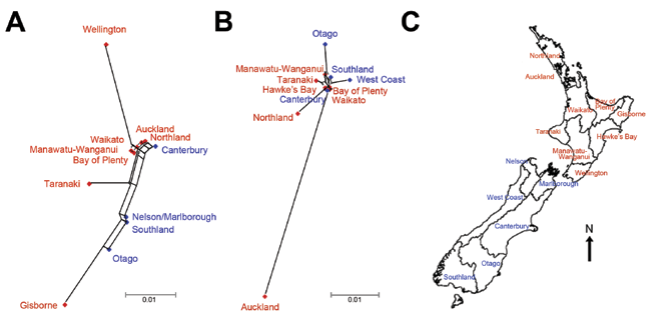
NeighborNet (*16*) trees showing population differentiation of Shiga toxin–producing *Escherichia coli* O157:H7 isolates from humans and cattle from different regions in the North Island (red) and the South Island (blue), New Zealand. A) Isolates from human case-patients (n = 355, 8 isolates excluded). B) Isolates from bovine meat samples (n = 233, 2 isolates excluded). C) Map of New Zealand showing different regions from which samples were collected. The distances indicate population differentiation measured as pairwise F_ST_ values.

χ^2^ and Fisher exact test for count data were used to evaluate associations between island and SBI type (AY2a, WY12a, ASY2c/SY2c, and other SBI types) for bovine fecal, bovine meat, and human isolates; R software (http://www.r-project.org/) was used for statistical computing. p values for associations between SBI types and region and between SBI types and year for human and bovine meat isolates were computed by simulating 10^8^ tables from the null hypothesis (independence) and comparing the results with the test statistic from the observed data.

Population differentiation among human and bovine meat isolates was assessed by using analysis of molecular variance (AMOVA) applied to haplotypes of isolates’ PFGE profiles (generated in BioNumerics) using Arlequin software version 3.5.1.2 (http://cmpg.unibe.ch/software/arlequin3/). A multilevel hierarchy was used for the AMOVA model to assess population differentiation between island, between regions within island, and within regions. Regions with <5 isolates were excluded from the analysis. A matrix of pairwise F_ST_ values was computed by comparing the PFGE haplotype frequency distributions for each pair of regions (using Arlequin, version 3.5.1.2). F_ST_ is an index of population differentiation, measuring the variance between subpopulations relative to the total variance, and ranges from 0 (no divergence) to 1 (complete divergence). The computed pairwise F_ST_ matrix, representing genetic distances between the regional populations of STEC O157:H7, was illustrated graphically as a NeighborNet tree by using SplitsTree software version 4.12.6 (*16*).

To illustrate the molecular relatedness and genotypic clustering of isolates, we used Primer 6 software (http://www.primer-e.com/primer.htm) to link distance matrices of PFGE profiles of human and bovine meat isolates (generated in BioNumerics) with explanatory variables (SBI type and region) to create multidimensional scaling plots. Regions with <5 isolates were excluded from the analysis.

To assess the population structure of New Zealand isolates, we compared published frequency distributions of SBI types in 205 cattle and 79 human STEC O157:H7 isolates sourced from Australia and in 143 cattle and 179 human STEC O157:H7 isolates sourced from the United States (*17*) with frequency distributions of SBI types among bovine and human STEC O157:H7 isolates from New Zealand. To evaluate genetic similarities of human and bovine fecal isolates, we computed proportional similarity indices (PSI) based on the frequency distributions of SBI types in humans and cattle from all 3 countries. PSI is a similarity measure that estimates the area of congruence between 2 frequency distributions (*18*); measurements range from 0 (distributions with no common SBI types) to 1 (highest possible similarity between distributions). Bootstrapped 95% confidence intervals for PSI values were calculated according to the percentile method described by Efron and Tibshirani (*19*), using 2,000 iterations. No grouping of SBI types was applied for PSI calculations. To illustrate the international geographic divergence of isolates, we used differences in PSI values (1 − PSI) to construct a NeighborNet tree with SplitsTree software version 4.12.6.

## Results

### Genotype Diversity

All 403 human and bovine fecal isolates were positive for *ehx*A, *eae*, and *stx*2 (except 1*chx*A-negative human isolate); of these, 61 (15.1%) were also positive for *stx*1. The different virulence profiles of isolates, each represented by a dominant SBI type, are shown in Table 1. The predominant SBI types AY2a, WY12a, and ASY2c/SY2c accounted for 55.0% (22/40), 15.0% (6/40), and 22.5% (9/40) of the studied bovine fecal isolates, respectively. Similarly, in human isolates, SBI types AY2a, WY12a, and ASY2c/SY2c were detected in 57.9% (210/363), 13.8% (50/363), and 23.1% (84/363) of the isolates, respectively. The distributions of AY2a, WY12a, ASY2c/SY2c, and other SBI types varied by year (p = 0.037) (Figure 1). On the basis of the genotype calibration of PFGE profiles of bovine meat isolates, SBI-like types AY2a, WY12a, and ASY2c/SY2c were prevalent in 64.7% (152/235), 23.4% (55/235), and 11.9% (28/235) of the isolates, respectively. Association between SBI-like type and year was marginally nonsignificant (p = 0.052).

**Table 1:** Virulence profiles and SBI types of Shiga toxin–producing *Escherichia coli* O157:H7 isolates obtained from humans and fecal samples from slaughterhouse cattle, New Zealand, 2008–2011*

Species, no. isolates	NI	SI	Virulence genes†						SBI type	
			*ehx*A	*eae*	*stx*2	*stx*2c	*stx*1		Dominant (no., %)	Other (no., %)
Bovine										
6	6	0	+	+	+	−	+		WY12a (6, 100.0)	–
10	2	8	+	+	+	+	−		ASY2c (7, 70.0), SY2c (2, 20.0)	AS2c (1, 10.0)
24	24	0	+	+	+	−	−		AY2a (22, 91.7)	AWY2a (2, 8.3)
Human										
51	43	9	+	+	+	–	+		WY12a (49, 96.2)	AWY12a (2, 3.8)
1	0	1	+	+	+	–	+		WY12a (1, 100.0)	–
94	54	40	+	+	+	+	−		ASY2c (69, 73.4), SY2c (15, 16.0)	SWY2c (3, 3.2), ASWY2c (2, 2.1), AS2c (2, 2.1), Y2c (2, 2.1), ASY2a2c (1, 1.1)
214	179	35	+	+	+	−	−		AY2a (210, 98.1)	AWY2a (4, 1.9)
3	2	1	+	+	+	+	+		ASY12c (2, 66.7)	AS12c (1, 33.3)

*NI, North Island of New Zealand; SBI, Shiga toxin–encoding bacteriophage insertion; SI, South Island of New Zealand; +, gene present; −, gene absent. †*ehx*A gene encodes for enterohemolysin; *eae* gene encodes for intimin; *stx*2, primers for detection of this gene did not differentiate between subtypes of Shiga toxin type 2; *stx*2c gene encodes for Shiga toxin subtype 2c; *stx*1, primers for detection of this gene did not differentiate between subtypes of Shiga toxin type 1.

### Between-Island Comparisons

The distribution of SBI types observed differed between North and South Islands in bovine fecal and human isolates; SBI types AY2a and WY12a were more common in the North Island, and ASY2c/SY2c was more common in the South Island (Table 2). Similarly, a significant difference in the prevalence of SBI-like types between islands was observed in bovine meat isolates (Table 2).

**Table 2:** Frequency distribution of predominant SBI genotypes of Shiga toxin–producing *Escherichia coli* O157:H7 isolates obtained from humans, bovine fecal samples, and bovine meat samples, New Zealand, 2008–2011*

Isolate type, SBI type	No. with SBI type/no. total (%)		p value†
	North Island	South Island	
Human			
AY2a	175/278 (62.9)	35/85 (41.2)	<0.001
WY12a	41/278 (14.7)	9/85 (10.6)	
ASY2c/SY2c	49/278 (17.6)	35/85 (41.2)	
Other	13/278 (4.7)	6/85 (7.1)	
Bovine fecal			
AY2a	22/32 (68.8)	0/8	<0.001
WY12a	6/32 (18.8)	0/8	
ASY2c/SY2c	1/32 (3.1)	8/8 (100.0)	
Bovine meat			
AY2a-like	137/201 (68.2)	15/34 (44.1)	<0.001
WY12a-like	49/201 (24.4)	6/34 (17.6)	
ASY2c/SY2c-like	15/201 (7.5)	13/34 (38.2)	

*SBI, Shiga toxin–encoding bacteriophage insertion. †Values refer to differences between frequency distributions of SBI types and North and South Islands (χ^2^ and Fisher exact test).

### Within-Island Comparisons

By using a 3-level hierarchy of island, region within island, and within region for the AMOVA model, we found that most of the molecular variation (>98%) resided between isolates within regions (on the basis of PFGE haplotypes). However, for the human isolates, a small but highly significant proportion of the molecular variation was estimated to be between regions within islands (1.03% variation, p<0.001); this finding provided evidence for highly localized geographic structuring. After we allowed for between region variation in the model, island was no longer a significant source of variation for the human isolates (p = 0.212). In contrast, a very small but significant amount of molecular variation was apparent between islands among the bovine meat isolates (0.38% variation, p = 0.017), but the proportion of variation between regions within islands was nonsignificant (0.34% variation, p = 0.121).

The population differentiation and geographic clustering of genotypes of STEC O157:H7 isolates from human cases and bovine meat samples from regions of both islands of New Zealand are illustrated in Figure 2. Consistent with the AMOVA results, we found evidence of within-island clustering of human isolates. Two main clusters were observed representing North and South Island regions, with the exception of Canterbury, which clustered with North Island regions, and Wellington, Taranaki, and Gisborne, which were North Island outliers. Among human cases, the highest population differentiation of genotypes of STEC O157:H7 isolates was observed between the regions of Wellington (15 isolates) and Gisborne (12 isolates) on the North Island (pairwise F_ST_ value of 0.071), followed by Wellington and Otago (10 isolates) (F_ST_ = 0.060); the isolates from Gisborne included 2 household clusters (2 human cases each). For bovine meat isolates, no obvious structuring was apparent; however, Auckland region (5 isolates) appeared as a strong North Island outlier. Consequently, the most distinct difference in genotypes was observed between the regions of Auckland and Otago (9 isolates) (F_ST_ = 0.060), followed by Auckland and Northland (26 isolates) (F_ST_ = 0.057).

**Figure 3:**
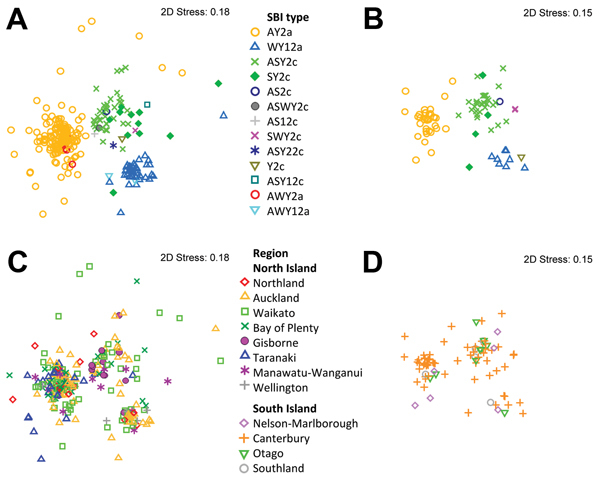
Multidimensional scaling plots showing the genotypic clustering of human Shiga toxin–producing *Escherichia coli* O157:H7 isolates originating from the North Island (n = 274, 4 isolates excluded) and the South Island (n = 81, 4 isolates excluded), New Zealand. The plots were determined on the basis of the isolates’ pulsed-field gel electrophoresis profiles. Clusters associated with Shiga toxin–encoding bacteriophage insertion (SBI) types (A) and regions (C) for isolates from the North Island. Clusters associated with SBI types (B) and regions (D) for isolates from the South Island. 2D, 2 dimensional.

The molecular relatedness between PFGE profiles of human isolates, considering SBI type and region of origin as explanatory variables, is shown in Figure 3. PFGE profiles showed genotypic clustering that was strongly associated with SBI types AY2a, WY12a, and ASY2c/SY2c, even after stratifying by island of origin (Figure 3, panels A, B). Clusters containing SBI type AY2a and ASY2c/SY2c were the predominant genotypes in the Taranaki and Gisborne regions, respectively, on the North Island (Figure 3, panel C); the association between SBI type and region of origin was statistically significant (p<0.001). A similar genotypic clustering of regions was observed in bovine meat isolates from the North and South Islands (Technical Appendix Figure 3).

### International Comparison

**Figure 4:**
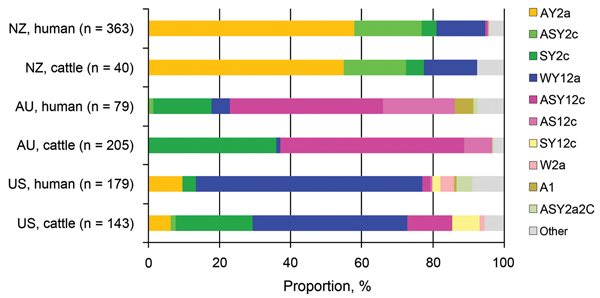
Proportional distributions of Shiga toxin–encoding bacteriophage insertion types of Shiga toxin–producing *Escherichia coli* O157:H7 isolates sourced from cattle and humans in New Zealand (NZ), Australia (AU), and the United States (US).

**Figure 5:**
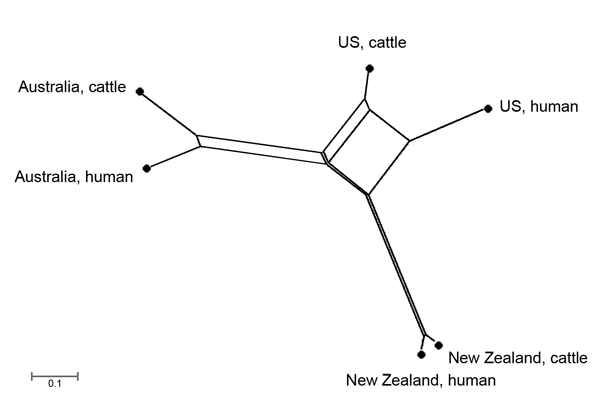
NeighborNet (*16*) tree showing geographic divergence of bovine and human Shiga toxin–producing *Escherichia coli* O157:H7 isolates sourced from New Zealand (40 cattle, 363 human), Australia (205 cattle, 79 human), and the United States (US) (143 cattle, 179 human). The distance indicates the difference in proportional similarity of Shiga toxin–encoding bacteriophage insertion types among the isolates.

Within each country, similar frequencies of SBI types were observed in cattle and human cases, but there were distinct differences in the population structure of SBI types between countries (Figure 4). Bovine and human genotypes in New Zealand shared the highest similarity (PSI value 0.92, 95% CI 0.74–0.93), followed by those in Australia (PSI 0.69, 95% CI 0.57–0.79) and the United States (PSI 0.61, 95% CI 0.51–0.69) (Technical Appendix Figure 4). The observed differences in proportional similarities of SBI types among isolates from cattle and humans in all 3 countries are shown in Figure 5.

## Discussion

We assessed the molecular epidemiologic evidence for transmission of STEC from cattle to humans in New Zealand and the relationship between population structure and geography at multiple spatial scales. The molecular analysis of bovine and human STEC O157:H7 isolates showed a concordant geographic variation of genotypes (SBI types) in both populations. In addition, there were marked differences between isolates from New Zealand’s North and South Islands, a finding that is consistent with localized transmission of STEC between cattle and humans.

The evidence of localized transmission of STEC between cattle and humans in New Zealand has advanced our understanding of the epidemiology of sporadic STEC infections in the country and is consistent with environmental- or animal-associated sources of infection rather than more disseminated foodborne outbreaks (*20*). Measures to prevent direct contact with animal fecal material in the environment include the wearing of protective clothing, increased hand washing, and targeted education of the population at risk regarding possible sources of STEC infection.

The North and South Islands of New Zealand are separated by the Cook Strait, a geographic barrier of >20 km. This barrier might contribute to the island-associated differences in distribution of genotypes observed in this study, by restricting the movement of carrier animals between islands. Cattle populations on each island are large: ≈6.6 million on the North Island and ≈3.5 million on the South Island (*21*). Despite the islands’ large cattle populations, the number of livestock moved between the islands (i.e., from farm to farm or farm to slaughter) is relatively low: ≈42,400 cattle from North to South Island, and ≈64,600 cattle from South to North Island per year (*22*). Thus, the movement of cattle probably has a limited influence on the distinct distribution of genotypes across both islands.

Although none of the bovine meat isolates were SBI typed, the PFGE data showed a strong island-associated distribution of bovine STEC O157:H7 genotypes, which was equivalent to the patterns observed in fecal isolates from cattle and humans. Bovine meat isolates were retrieved from carcass swab samples and bulk meat samples collected at beef-processing plants, so it could be hypothesized that fresh beef meat might be an exposure pathway for humans. However, although various food sources (including beef) were considered as potential risk factors during a nationwide prospective case-control study on sporadic STEC infections in humans, food was not identified as a major exposure pathway of infections in New Zealand (*20*).

Significant genetic variation was observed among human isolates at the regional level, indicating a more localized spatial clustering of STEC O157:H7 genotypes. Strong regional variation in the prevalence of zoonotic diseases has been observed previously in New Zealand. For example, there is marked regional variation in the distribution of serotypes in human cases of salmonellosis: *Salmonella enterica* ser. Brandenburg was associated with sheep and human infections in the southern regions of the South Island (*23*), whereas the wild bird–associated *S. enterica* ser. Typhimurium DT160 was distributed more evenly across the whole country (*24*). *S. enterica* ser. Brandenburg has not been found to be endemic in any other regions in New Zealand, and it is likely that the spatial pattern of disease is influenced by environmental factors, such as the presence and density of local maintenance hosts.

Cattle are considered the most likely maintenance host of STEC O157:H7, and the association between human cases and cattle density suggests that spillover from cattle to humans is the main pathway (*20*); however, overseas, the pathogen has frequently been isolated from sheep (*25*–*27*) and deer (*28*,*29*). Cookson et al. (*30*,*31*) identified STEC serotypes of public health concern in sheep from the lower North Island of New Zealand but did not isolate STEC O157:H7. No nationwide studies of sheep or deer have been undertaken in New Zealand, hence sheep cannot be ruled out as potential maintenance hosts for region-specific populations of STEC O157:H7.

The observed regional clustering of genotypes among human STEC O157:H7 isolates leads to another hypothesis: other, yet unidentified, hosts could be reservoir/maintenance hosts in the epidemiology of STEC, and cattle are possibly only serving as “bridging hosts” at the human–animal interface (*32*), transmitting STEC to humans. For example, starlings have been implicated as biologic vectors in the dissemination of STEC among dairy farms in Ohio, United States (*33*,*34*), indicating that wildlife might play a key role in the epidemiology and ecology of STEC.

A relatively high prevalence of SBI types AY2a and ASY2c was observed in human and bovine fecal isolates from New Zealand. These findings are in contrast to those from the Australian study by Mellor et al. (*17*), in which SBI type AY2a was not identified (0/284 isolates; p<0.001) and accounted for only 8.1% (26/322) of the isolates from the United States (human and cattle combined); SBI type ASY2c was prevalent in <1.0% of combined isolates in both countries. These differences in frequency distributions of SBI types indicate marked differences in the population structure of SBI types between countries. Australia and New Zealand are neighboring countries but separated by the Tasman Sea, a distance of ≈1,250 km. On the basis of historic data, Australia has been the predominant source of imported New Zealand cattle, mainly in the 19th century (*5*). Hence, the distinct geographic divergence of STEC O157:H7 genotypes between the 2 countries is somewhat puzzling and would suggest a limited historic introduction of STEC O157:H7 from Australia or elsewhere into New Zealand and a subsequent evolution in the New Zealand host population. Alternatively, the observed divergence of genotypes between Australia and New Zealand could be the result of genetic drift and/or selection driven by different environmental factors, such as climate, types of feed, husbandry systems, or animal genetics.

In this study, the highest PSI was observed between cattle and human isolates from New Zealand, followed by that between isolates from Australia and the United States. These findings provide evidence for a close association between populations of isolates from cattle and humans, which is consistent with the transmission of STEC from cattle to humans. This finding is in agreement with the national case–control study on clinical STEC cases in New Zealand, which identified variables related to beef and dairy cattle as major risk factors (*20*).

The molecular analysis of STEC O157:H7 isolates from cattle and persons with STEC infection revealed that prevalences of bovine and human isolates in the North Island were distinctly different from those of the South Island, suggesting localized transmission of STEC between cattle and humans. Furthermore, a distribution of STEC O157:H7 genotypes different from that observed overseas suggests a historic introduction of a subset of the globally circulating STEC O157:H7 strains into New Zealand.

Technical Appendix Pulsed-field gel electrophoresis profiles of human and bovine *Escherichia coli* O157:H7 isolates, multidimensional scaling plots showing genotypic clustering of isolates, and proportional similarity index values.
