# Supplementary material for: Geographic Divergence of Bovine and Human Shiga Toxin–Producing Escherichia coli O157:H7 Genotypes, New Zealand
Source: Emerg Infect Dis. 2014 Dec;20(12):1980–9. doi: 10.3201/eid2012.140281 (PMC4257794; doi:10.3201/eid2012.140281)
Supplement: Technical Appendix — Pulsed-field gel electrophoresis profiles of human and bovine Escherichia coli O157:H7 isolates, multidimensional scaling plots showing genotypic clustering of isolates, and proportional similarity index values. [file 14-0281-Techapp-s1.pdf]

Article DOI: http://dx.doi.org/10.3201/eid2012.140281

Geographic Divergence of Bovine and Human Shiga Toxin–Producing *Escherichia coli* O157:H7 Genotypes, New Zealand

Technical Appendix

Technical Appendix Figure 1 (below and following pages). PFGE profile comparison of 363 human Shiga toxin–producing *Escherichia coli* O157:H7 isolates using UPGMA cluster analysis (Dice similarity coefficient, 1% band matching tolerance). Virulence genes (*stx*1, *stx*2a/2c, *stx*2c, *eae*, *ehx*A) present (1) or absent (0). Island of origin (Origin) presented as North Island (NI) or South Island (SI) of New Zealand, and genotypes of isolates as Shiga toxin (Stx)-encoding bacteriophage insertion (SBI) types.

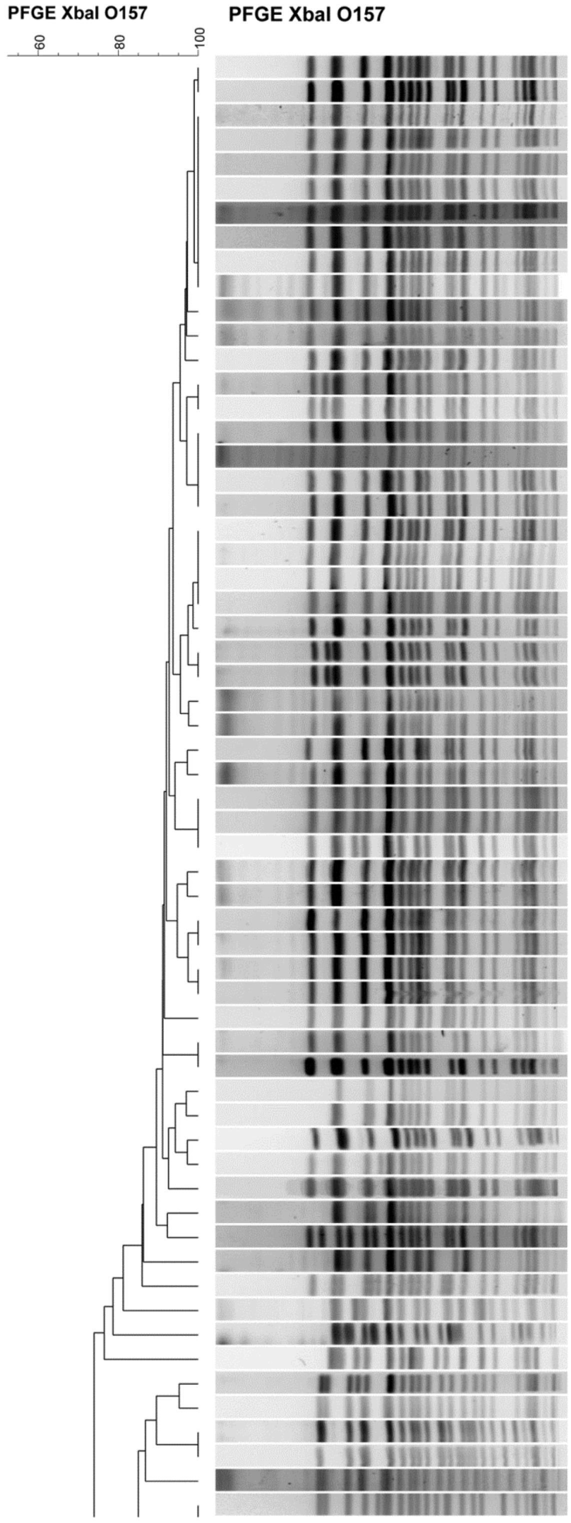

Strain	stx1	stx2a/2c	stx2c	eae	ehxA	Origin	SBI type
EcHu183a	1	1	0	1	1	NI	WY12a
EcHu331a	1	1	0	1	1	NI	WY12a
EcHu86a	1	1	0	1	1	NI	WY12a
EcHu177a	1	1	0	1	1	NI	WY12a
EcHu190a	1	1	0	1	1	NI	WY12a
EcHu196a	1	1	0	1	1	NI	WY12a
EcHu429a	1	1	0	1	1	SI	WY12a
EcHu95a	1	1	0	1	1	NI	WY12a
EcHu153a	1	1	0	1	1	NI	WY12a
EcHu99a	1	1	0	1	1	NI	WY12a
EcHu217a	1	1	0	1	1	NI	WY12a
EcHu420a	1	1	0	1	1	NI	WY12a
EcHu257a	1	1	0	1	0	SI	WY12a
EcHu306a	1	1	0	1	1	NI	WY12a
EcHu120a	1	1	0	1	1	NI	WY12a
EcHu277a	1	1	0	1	1	NI	WY12a
EcHu85a	1	1	0	1	1	NI	WY12a
EcHu224a	1	1	0	1	1	NI	WY12a
EcHu397a	1	1	0	1	1	NI	WY12a
EcHu207a	1	1	0	1	1	NI	WY12a
EcHu272a	1	1	0	1	1	NI	WY12a
EcHu157a	1	1	0	1	1	SI	WY12a
EcHu161a	1	1	0	1	1	SI	WY12a
EcHu447a	1	1	0	1	1	SI	WY12a
EcHu439a	1	1	0	1	1	SI	WY12a
EcHu440a	1	1	0	1	1	SI	WY12a
EcHu275a	1	1	0	1	1	SI	WY12a
EcHu278a	1	1	0	1	1	SI	WY12a
EcHu110a	1	1	0	1	1	NI	WY12a
EcHu276a	1	1	0	1	1	NI	WY12a
EcHu158a	1	1	0	1	1	NI	WY12a
EcHu310a	1	1	0	1	1	NI	WY12a
EcHu163a	1	1	0	1	1	NI	WY12a
EcHu362a	1	1	0	1	1	NI	WY12a
EcHu374a	1	1	0	1	1	NI	WY12a
EcHu376a	1	1	0	1	1	NI	AWY12a
EcHu379a	1	1	0	1	1	NI	WY12a
EcHu372a	1	1	0	1	1	NI	WY12a
EcHu373a	1	1	0	1	1	NI	WY12a
EcHu262a	1	1	0	1	1	NI	WY12a
EcHu377a	1	1	0	1	1	NI	WY12a
EcHu355a	1	1	0	1	1	NI	WY12a
EcHu87a	1	1	0	1	1	NI	WY12a
EcHu286a	1	1	0	1	1	NI	WY12a
EcHu324a	1	1	0	1	1	NI	WY12a
EcHu135a	1	1	0	1	1	NI	WY12a
EcHu460a	1	1	0	1	1	NI	WY12a
EcHu249a	1	1	0	1	1	NI	WY12a
EcHu387a	1	1	0	1	1	NI	AWY12a
EcHu232a	1	1	0	1	1	NI	WY12a
EcHu93a	0	1	1	1	1	SI	Y2c
EcHu117a	0	1	1	1	1	NI	SY2c
EcHu451a	1	1	0	1	1	NI	WY12a
EcHu168a	0	1	1	1	1	NI	SY2c
EcHu428a	0	1	0	1	1	SI	AY2a
EcHu291a	0	1	0	1	1	NI	AY2a
EcHu449a	0	1	0	1	1	NI	AY2a
EcHu125a	0	1	0	1	1	NI	AY2a
EcHu84a	0	1	0	1	1	NI	AY2a
EcHu185a	0	1	0	1	1	SI	AY2a

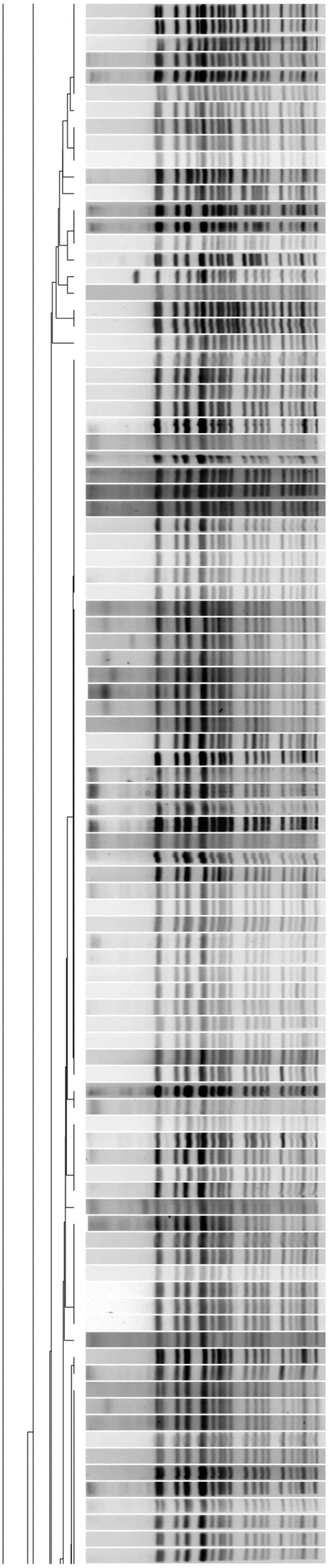

EcHu330a	0	1	0	1	1	NI	AY2a
EcHu333a	0	1	0	1	1	NI	AY2a
EcHu417a	0	1	0	1	1	NI	AY2a
EcHu441a	0	1	0	1	1	NI	AY2a
EcHu442a	0	1	0	1	1	NI	AY2a
EcHu294a	0	1	0	1	1	NI	AY2a
EcHu270a	0	1	0	1	1	NI	AY2a
EcHu261a	0	1	0	1	1	NI	AY2a
EcHu285a	0	1	0	1	1	NI	AY2a
EcHu298a	0	1	0	1	1	NI	AY2a
EcHu401a	0	1	0	1	1	NI	AY2a
EcHu181a	0	1	0	1	1	NI	AY2a
EcHu357a	0	1	0	1	1	NI	AY2a
EcHu358a	0	1	0	1	1	SI	AY2a
EcHu131a	0	1	0	1	1	SI	AY2a
EcHu408a	0	1	0	1	1	NI	AY2a
EcHu325a	0	1	0	1	1	NI	AY2a
EcHu274a	0	1	0	1	1	NI	AY2a
EcHu202a	0	1	0	1	1	NI	AY2a
EcHu203a	0	1	0	1	1	NI	AY2a
EcHu200a	0	1	0	1	1	NI	AY2a
EcHu231a	0	1	0	1	1	NI	AY2a
EcHu150a	0	1	0	1	1	NI	AY2a
EcHu233a	0	1	0	1	1	SI	AY2a
EcHu205a	0	1	0	1	1	NI	AY2a
EcHu384a	0	1	0	1	1	SI	AY2a
EcHu419a	0	1	0	1	1	NI	AY2a
EcHu353a	0	1	0	1	1	NI	AY2a
EcHu380a	0	1	0	1	1	NI	AY2a
EcHu383a	0	1	0	1	1	SI	AY2a
EcHu434a	0	1	0	1	1	SI	AY2a
EcHu448a	0	1	0	1	1	NI	AY2a
EcHu269a	0	1	0	1	1	SI	AY2a
EcHu104a	0	1	0	1	1	NI	AY2a
EcHu118a	0	1	0	1	1	NI	AY2a
EcHu128a	0	1	0	1	1	NI	AY2a
EcHu214a	0	1	0	1	1	NI	AY2a
EcHu218a	0	1	0	1	1	NI	AY2a
EcHu226a	0	1	0	1	1	NI	AY2a
EcHu228a	0	1	0	1	1	NI	AY2a
EcHu241a	0	1	0	1	1	NI	AWY2a
EcHu244a	0	1	0	1	1	NI	AY2a
EcHu256a	0	1	0	1	1	NI	AY2a
EcHu316a	0	1	0	1	1	NI	AY2a
EcHu184a	0	1	0	1	1	NI	AY2a
EcHu334a	0	1	0	1	1	NI	AY2a
EcHu390a	0	1	0	1	1	NI	AY2a
EcHu391a	0	1	0	1	1	SI	AY2a
EcHu392a	0	1	0	1	1	SI	AY2a
EcHu395a	0	1	0	1	1	SI	AY2a
EcHu422a	0	1	0	1	1	NI	AY2a
EcHu352a	0	1	0	1	1	NI	AY2a
EcHu446a	0	1	0	1	1	NI	AY2a
EcHu98a	0	1	0	1	1	NI	AY2a
EcHu100a	0	1	0	1	1	SI	AY2a
EcHu264a	0	1	0	1	1	NI	AY2a
EcHu284a	0	1	0	1	1	NI	AY2a
EcHu290a	0	1	0	1	1	NI	AY2a
EcHu292a	0	1	0	1	1	SI	AY2a
EcHu113a	0	1	0	1	1	NI	AY2a
EcHu116a	0	1	0	1	1	NI	AY2a
EcHu136a	0	1	0	1	1	NI	AY2a
EcHu137a	0	1	0	1	1	SI	AY2a
EcHu160a	0	1	0	1	1	NI	AY2a
EcHu164a	0	1	0	1	1	NI	AY2a
EcHu356a	0	1	0	1	1	NI	AY2a
EcHu103a	0	1	0	1	1	NI	AY2a
EcHu194a	0	1	0	1	1	SI	AY2a
EcHu361a	0	1	0	1	1	SI	AY2a
EcHu371a	0	1	0	1	1	NI	AY2a
EcHu126a	0	1	0	1	1	NI	AY2a
EcHu370a	0	1	0	1	1	SI	AY2a
EcHu421a	0	1	0	1	1	NI	AY2a
EcHu88a	0	1	0	1	1	NI	AY2a
EcHu176a	0	1	0	1	1	NI	AY2a
EcHu179a	0	1	0	1	1	SI	AY2a
EcHu129a	0	1	0	1	1	SI	AY2a
EcHu171a	0	1	0	1	1	NI	AY2a
EcHu172a	0	1	0	1	1	NI	AY2a
EcHu173a	0	1	0	1	1	NI	AY2a
EcHu147a	0	1	0	1	1	NI	AY2a
EcHu400a	0	1	0	1	1	NI	AY2a
EcHu405a	0	1	0	1	1	NI	AY2a
EcHu197a	0	1	0	1	1	NI	AY2a
EcHu255a	0	1	0	1	1	NI	AY2a
EcHu317a	0	1	0	1	1	NI	AY2a
EcHu247a	0	1	0	1	1	NI	AY2a
EcHu188a	0	1	0	1	1	NI	AY2a
EcHu339a	0	1	0	1	1	NI	AY2a
EcHu404a	0	1	0	1	1	NI	AY2a
EcHu423a	0	1	0	1	1	NI	AY2a
EcHu427a	0	1	0	1	1	NI	AY2a
EcHu348a	0	1	0	1	1	NI	AY2a
EcHu349a	0	1	0	1	1	NI	AY2a

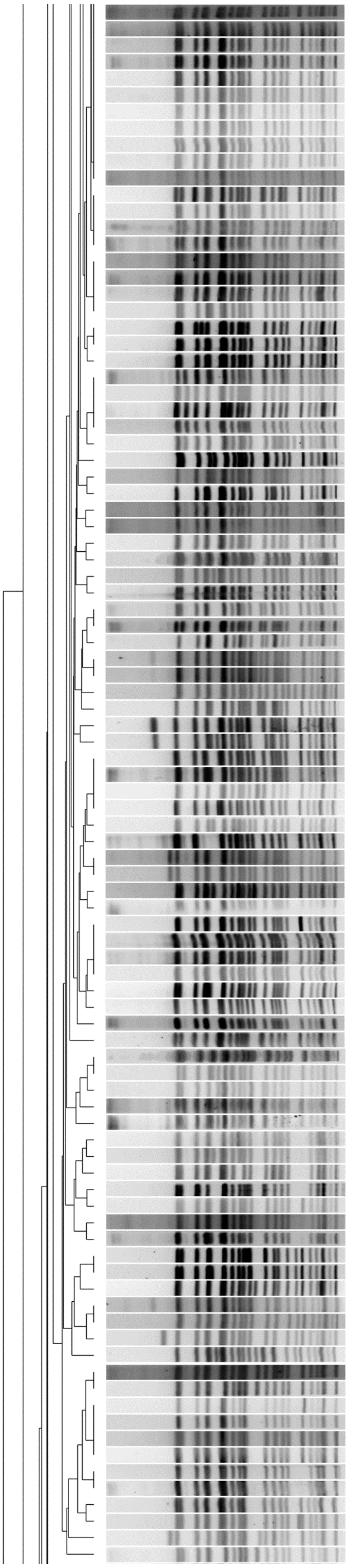

EcHu382a	0	1	0	1	1	NI	AY2a
EcHu436a	0	1	0	1	1	SI	AY2a
EcHu443a	0	1	0	1	1	NI	AY2a
EcHu445a	0	1	0	1	1	NI	AY2a
EcHu455a	0	1	0	1	1	NI	AY2a
EcHu267a	0	1	0	1	1	NI	AY2a
EcHu111a	0	1	0	1	1	NI	AY2a
EcHu119a	0	1	0	1	1	NI	AY2a
EcHu305a	0	1	0	1	1	NI	AY2a
EcHu307a	0	1	0	1	1	NI	AY2a
EcHu138a	0	1	0	1	1	NI	AY2a
EcHu182a	0	1	0	1	1	NI	AY2a
EcHu454a	0	1	0	1	1	NI	AY2a
EcHu192a	0	1	0	1	1	NI	AY2a
EcHu402a	0	1	0	1	1	NI	AY2a
EcHu403a	0	1	0	1	1	NI	AY2a
EcHu388a	0	1	0	1	1	NI	AY2a
EcHu396a	0	1	0	1	1	NI	AY2a
EcHu296a	0	1	0	1	1	SI	AY2a
EcHu344a	0	1	0	1	1	NI	AY2a
EcHu416a	0	1	0	1	1	NI	AY2a
EcHu415a	0	1	0	1	1	NI	AY2a
EcHu406a	0	1	0	1	1	NI	AY2a
EcHu268a	0	1	0	1	1	NI	AY2a
EcHu367a	0	1	0	1	1	NI	AY2a
EcHu438a	0	1	0	1	1	NI	AY2a
EcHu239a	0	1	0	1	1	SI	AY2a
EcHu341a	0	1	0	1	1	NI	AY2a
EcHu146a	0	1	0	1	1	SI	AY2a
EcHu336a	0	1	0	1	1	NI	AY2a
EcHu141a	0	1	0	1	1	NI	AY2a
EcHu142a	0	1	0	1	1	NI	AY2a
EcHu238a	0	1	0	1	1	NI	AY2a
EcHu459a	0	1	0	1	1	NI	AY2a
EcHu187a	0	1	0	1	1	NI	AY2a
EcHu413a	0	1	0	1	1	NI	AY2a
EcHu426a	0	1	0	1	1	NI	AY2a
EcHu359a	0	1	0	1	1	NI	AY2a
EcHu350a	0	1	0	1	1	NI	AY2a
EcHu234a	0	1	0	1	1	SI	AY2a
EcHu91a	0	1	0	1	1	SI	AY2a
EcHu193a	0	1	0	1	1	NI	AY2a
EcHu144a	0	1	0	1	1	NI	AY2a
EcHu206a	0	1	0	1	1	NI	AY2a
EcHu149a	0	1	0	1	1	NI	AY2a
EcHu201a	0	1	0	1	1	NI	AY2a
EcHu389a	0	1	0	1	1	NI	AY2a
EcHu302a	0	1	0	1	1	NI	AY2a
EcHu155a	0	1	0	1	1	NI	AY2a
EcHu127a	0	1	0	1	1	SI	AY2a
EcHu410a	0	1	0	1	1	NI	AY2a
EcHu315a	0	1	0	1	1	NI	AWY2a
EcHu189a	0	1	0	1	1	NI	AWY2a
EcHu386a	0	1	0	1	1	NI	AY2a
EcHu289a	0	1	0	1	1	SI	AY2a
EcHu326a	0	1	0	1	1	NI	AY2a
EcHu411a	0	1	0	1	1	NI	AY2a
EcHu412a	0	1	0	1	1	NI	AY2a
EcHu122a	0	1	0	1	1	NI	AY2a
EcHu385a	0	1	0	1	1	SI	AY2a
EcHu407a	0	1	0	1	1	NI	AY2a
EcHu354a	0	1	0	1	1	SI	AY2a
EcHu204a	0	1	0	1	1	SI	AY2a
EcHu457a	0	1	0	1	1	SI	AY2a
EcHu297a	0	1	0	1	1	NI	AY2a
EcHu283a	0	1	0	1	1	NI	AY2a
EcHu223a	0	1	0	1	1	NI	AY2a
EcHu398a	0	1	0	1	1	NI	AY2a
EcHu213a	0	1	0	1	1	NI	AY2a
EcHu265a	0	1	0	1	1	NI	AY2a
EcHu308a	0	1	0	1	1	NI	AY2a
EcHu343a	0	1	0	1	1	SI	AY2a
EcHu134a	0	1	0	1	1	NI	AY2a
EcHu225a	0	1	0	1	1	NI	AY2a
EcHu444a	0	1	0	1	1	NI	AY2a
EcHu327a	0	1	0	1	1	NI	AY2a
EcHu329a	0	1	0	1	1	NI	AY2a
EcHu368a	0	1	0	1	1	SI	AY2a
EcHu253a	0	1	0	1	1	NI	AY2a
EcHu191a	0	1	0	1	1	NI	AY2a
EcHu263a	0	1	0	1	1	NI	AWY2a
EcHu450a	0	1	0	1	1	NI	AY2a
EcHu431a	0	1	0	1	1	NI	AY2a
EcHu151a	0	1	0	1	1	NI	AY2a
EcHu212a	0	1	0	1	1	NI	AY2a
EcHu347a	0	1	0	1	1	NI	AY2a
EcHu159a	0	1	0	1	1	NI	AY2a
EcHu167a	0	1	0	1	1	NI	AY2a
EcHu393a	0	1	0	1	1	NI	AY2a
EcHu394a	0	1	0	1	1	NI	AY2a
EcHu452a	0	1	0	1	1	NI	AY2a
EcHu266a	0	1	0	1	1	NI	AY2a
EcHu287a	0	1	0	1	1	SI	AY2a
EcHu96a	0	1	0	1	1	NI	AY2a

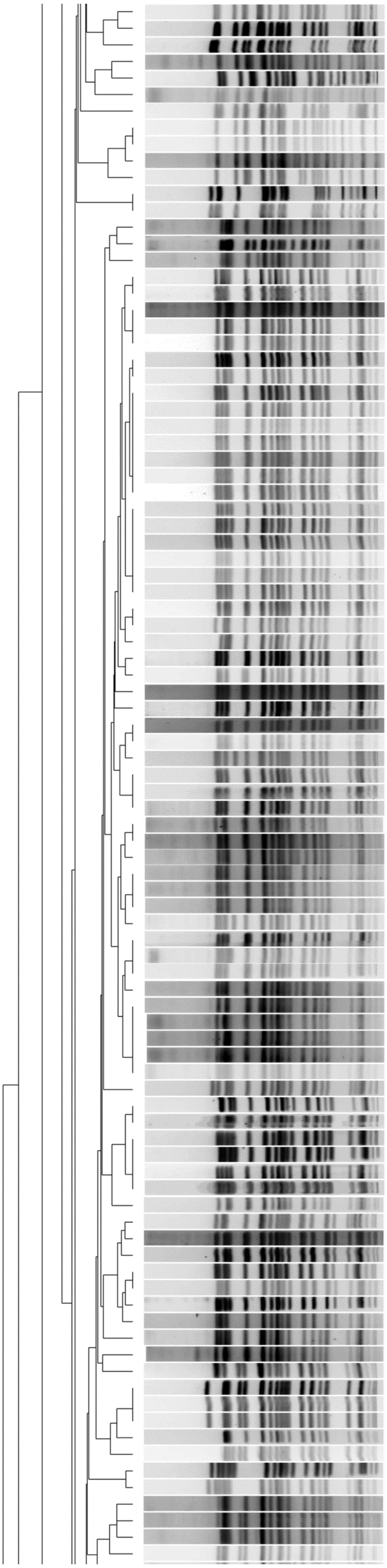

EcHu309a	0	1	0	1	1	NI	AY2a
EcHu328a	0	1	0	1	1	NI	AY2a
EcHu345a	0	1	0	1	1	NI	AY2a
EcHu235a	0	1	0	1	1	NI	AY2a
EcHu323a	0	1	0	1	1	NI	AY2a
EcHu279a	0	1	0	1	1	NI	AY2a
EcHu195a	0	1	0	1	1	NI	AY2a
EcHu300a	0	1	0	1	1	NI	AY2a
EcHu301a	0	1	0	1	1	NI	AY2a
EcHu211a	0	1	0	1	1	NI	AY2a
EcHu102a	0	1	0	1	1	NI	AY2a
EcHu342a	0	1	0	1	1	NI	AY2a
EcHu124a	0	1	0	1	1	NI	AY2a
EcHu166a	0	1	1	1	1	NI	SY2c
EcHu360a	0	1	1	1	1	NI	SY2c
EcHu260a	0	1	1	1	1	SI	SY2c
EcHu156a	0	1	1	1	1	SI	AS2c
EcHu165a	0	1	1	1	1	SI	ASY2c
EcHu433a	0	1	1	1	1	SI	ASY2c
EcHu140a	0	1	1	1	1	NI	ASY2c
EcHu170a	0	1	1	1	1	NI	ASY2c
EcHu332a	0	1	1	1	1	SI	ASY2c
EcHu121a	0	1	1	1	1	SI	ASY2c
EcHu198a	0	1	1	1	1	NI	ASY2c
EcHu123a	0	1	1	1	1	NI	AS2c
EcHu130a	0	1	1	1	1	SI	ASY2c
EcHu139a	0	1	1	1	1	SI	ASY2c
EcHu162a	0	1	1	1	1	NI	ASY2c
EcHu314a	0	1	1	1	1	SI	ASY2c
EcHu169a	0	1	1	1	1	SI	ASY2c
EcHu424a	0	1	1	1	1	SI	ASY2c
EcHu145a	0	1	1	1	1	NI	ASY2c
EcHu437a	0	1	1	1	1	SI	ASY2c
EcHu299a	0	1	1	1	1	SI	ASY2c
EcHu133a	0	1	1	1	1	SI	ASY2c
EcHu143a	0	1	1	1	1	NI	ASY2c
EcHu180a	0	1	1	1	1	NI	SY2c
EcHu271a	0	1	1	1	1	SI	ASY2c
EcHu273a	0	1	1	1	1	NI	SY2c
EcHu365a	0	1	1	1	1	SI	ASY2c
EcHu112a	0	1	1	1	1	NI	ASY2c
EcHu432a	0	1	1	1	1	NI	ASY2c
EcHu363a	0	1	1	1	1	NI	ASY2c
EcHu430a	0	1	1	1	1	SI	ASY2c
EcHu115a	0	1	1	1	1	SI	ASY2c
EcHu186a	0	1	0	1	1	NI	AY2a
EcHu92a	0	1	1	1	1	NI	ASY2c
EcHu208a	0	1	1	1	1	NI	ASY2c
EcHu97a	0	1	1	1	1	SI	ASY2c
EcHu242a	1	1	1	1	1	NI	AS12c
EcHu254a	0	1	1	1	1	NI	ASY2c
EcHu230a	0	1	1	1	1	NI	ASY2c
EcHu318a	0	1	1	1	1	NI	ASY2c
EcHu319a	0	1	1	1	1	NI	ASY2c
EcHu320a	0	1	1	1	1	NI	ASY2c
EcHu304a	0	1	1	1	1	SI	ASY2c
EcHu414a	0	1	1	1	1	NI	ASY2c
EcHu280a	0	1	1	1	1	SI	ASY2c
EcHu132a	0	1	1	1	1	NI	ASY2c
EcHu148a	0	1	1	1	1	NI	ASY2c
EcHu229a	0	1	1	1	1	SI	ASY2c
EcHu243a	0	1	1	1	1	NI	ASWY2c
EcHu245a	0	1	1	1	1	NI	ASY2c
EcHu251a	0	1	1	1	1	SI	ASY2c
EcHu101a	0	1	1	1	1	SI	ASY2c
EcHu178a	0	1	1	1	1	NI	ASY2c
EcHu340a	0	1	1	1	1	NI	ASY2c
EcHu456a	0	1	1	1	1	SI	ASY2c
EcHu209a	0	1	1	1	1	NI	ASY2c
EcHu322a	0	1	1	1	1	NI	ASY2c
EcHu418a	0	1	1	1	1	NI	ASY2c
EcHu458a	0	1	1	1	1	NI	ASY2c
EcHu351a	0	1	1	1	1	NI	ASY2c
EcHu227a	0	1	1	1	1	NI	ASY2c
EcHu381a	0	1	1	1	1	SI	ASY2c
EcHu399a	0	1	1	1	1	SI	ASY2c
EcHu453a	0	1	1	1	1	SI	ASY2c
EcHu295a	0	1	1	1	1	NI	ASY2c
EcHu335a	0	1	1	1	1	NI	ASY2c
EcHu94a	0	1	1	1	1	SI	ASY2c
EcHu375a	0	1	1	1	1	NI	ASY2c
EcHu246a	0	1	1	1	1	SI	ASY2c
EcHu369a	0	1	1	1	1	SI	ASY2c
EcHu346a	0	1	1	1	1	NI	SWY2c
EcHu312a	0	1	1	1	1	SI	SWY2c
EcHu313a	0	1	1	1	1	SI	SWY2c
EcHu425a	0	1	1	1	1	SI	SY2c
EcHu311a	0	1	1	1	1	SI	SY2c
EcHu321a	0	1	1	1	1	NI	ASY2c
EcHu288a	0	1	1	1	1	NI	ASY2c
EcHu215a	0	1	1	1	1	NI	ASY2c
EcHu216a	0	1	1	1	1	NI	ASY2c
EcHu219a	0	1	1	1	1	NI	SY2c
EcHu114a	0	1	1	1	1	SI	ASY2c

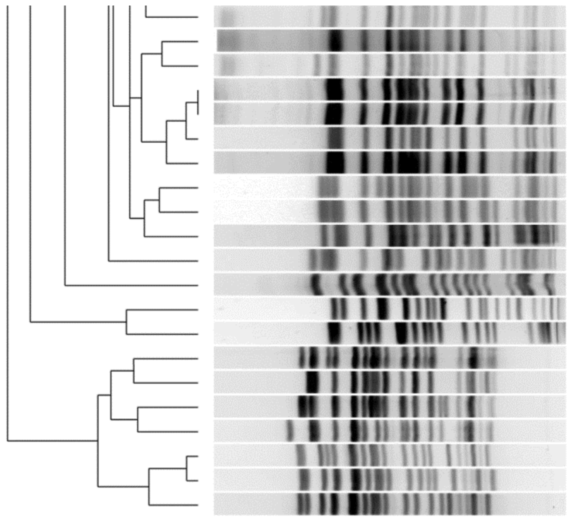

EcHu281a	0	1	1	1	1	NI	ASY2c
EcHu252a	0	1	1	1	1	NI	ASY2a2c
EcHu282a	0	1	1	1	1	SI	SY2c
EcHu364a	0	1	1	1	1	NI	SY2c
EcHu366a	0	1	1	1	1	NI	SY2c
EcHu152a	0	1	1	1	1	NI	Y2c
EcHu378a	0	1	1	1	1	NI	SY2c
EcHu175a	0	1	1	1	1	NI	ASY2c
EcHu174a	0	1	1	1	1	NI	ASY2c
EcHu210a	1	1	1	1	1	NI	ASY12c
EcHu258a	1	1	1	1	1	SI	ASY12c
EcHu409a	0	1	0	1	1	NI	AY2a
EcHu337a	0	1	0	1	1	NI	AY2a
EcHu338a	0	1	0	1	1	NI	AY2a
EcHu89a	0	1	1	1	1	SI	ASY2c
EcHu236a	0	1	1	1	1	NI	SY2c
EcHu248a	0	1	1	1	1	SI	ASWY2c
EcHu90a	1	1	0	1	1	NI	WY12a
EcHu250a	0	1	0	1	1	NI	AY2a
EcHu199a	0	1	0	1	1	NI	AY2a
EcHu259a	0	1	0	1	1	NI	AY2a

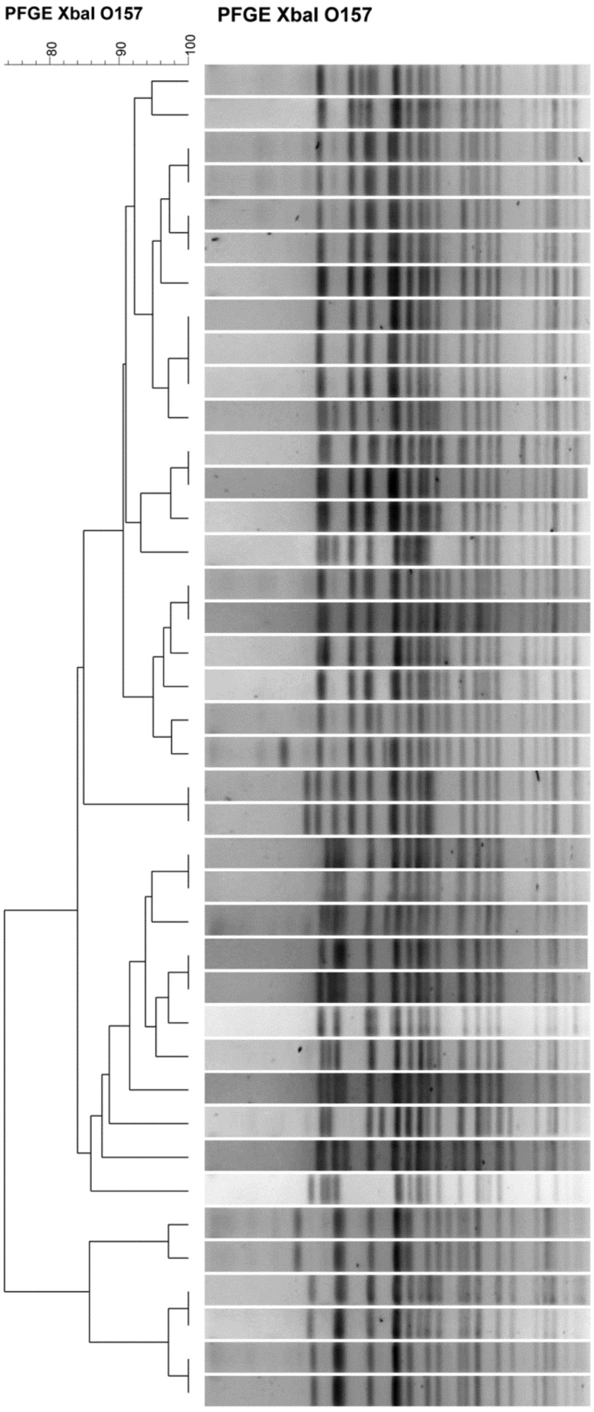

Key	stx1	stx2a/2c	stx2c	eae	ehxA	Origin	SBI type
EcCa129a	0	1	0	1	1	NI	AY2a
EcCa25a	0	1	0	1	1	NI	AY2a
EcCa126a	0	1	0	1	1	NI	AY2a
EcCa127a	0	1	0	1	1	NI	AY2a
EcCa124a	0	1	0	1	1	NI	AY2a
EcCa441a	0	1	0	1	1	NI	AY2a
EcCa439a	0	1	0	1	1	NI	AY2a
EcCa23a	0	1	0	1	1	NI	AY2a
EcCa30a	0	1	0	1	1	NI	AY2a
EcCa634a	0	1	0	1	1	NI	AY2a
EcCa443a	0	1	0	1	1	NI	AY2a
EcCa31a	0	1	0	1	1	NI	AY2a
EcCa491a	0	1	0	1	1	NI	AY2a
EcCa130a	0	1	0	1	1	NI	AY2a
EcCa28a	0	1	0	1	1	NI	AY2a
EcCa122a	0	1	0	1	1	NI	AY2a
EcCa572a	0	1	0	1	1	NI	AY2a
EcCa431a	0	1	0	1	1	NI	AY2a
EcCa27a	0	1	0	1	1	NI	AY2a
EcCa125a	0	1	0	1	1	NI	AY2a
EcCa128a	0	1	0	1	1	NI	AY2a
EcCa462a	0	1	0	1	1	NI	AWY2a
EcCa463a	0	1	0	1	1	NI	AWY2a
EcCa452a	0	1	1	1	1	SI	SY2c
EcCa474a	0	1	1	1	1	SI	SY2c
EcCa58a	0	1	1	1	1	SI	ASY2c
EcCa432a	0	1	1	1	1	SI	ASY2c
EcCa57a	0	1	1	1	1	SI	ASY2c
EcCa63a	0	1	0	1	1	NI	AY2a
EcCa636a	0	1	1	1	1	SI	ASY2c
EcCa34a	0	1	1	1	1	NI	AS2c
EcCa635a	0	1	1	1	1	SI	ASY2c
EcCa451a	0	1	1	1	1	SI	ASY2c
EcCa484a	0	1	1	1	1	NI	ASY2c
EcCa120a	1	1	0	1	1	NI	WY12a
EcCa121a	1	1	0	1	1	NI	WY12a
EcCa29a	1	1	0	1	1	NI	WY12a
EcCa32a	1	1	0	1	1	NI	WY12a
EcCa123a	1	1	0	1	1	NI	WY12a
EcCa461b	1	1	0	1	1	NI	WY12a

Technical Appendix Figure 2. PFGE profile comparison of 40 bovine Shiga toxin–producing *Escherichia coli* O157:H7 isolates using UPGMA cluster analysis (Dice similarity coefficient, 1% band matching tolerance). Virulence genes (*stx*1, *stx*2a/2c, *stx*2c, *eae*, *ehx*A) present (1) or absent (0). Island of origin (Origin) presented as North Island (NI) or South Island (SI) of New Zealand, and genotypes of isolates as Shiga toxin-encoding bacteriophage insertion (SBI) types.

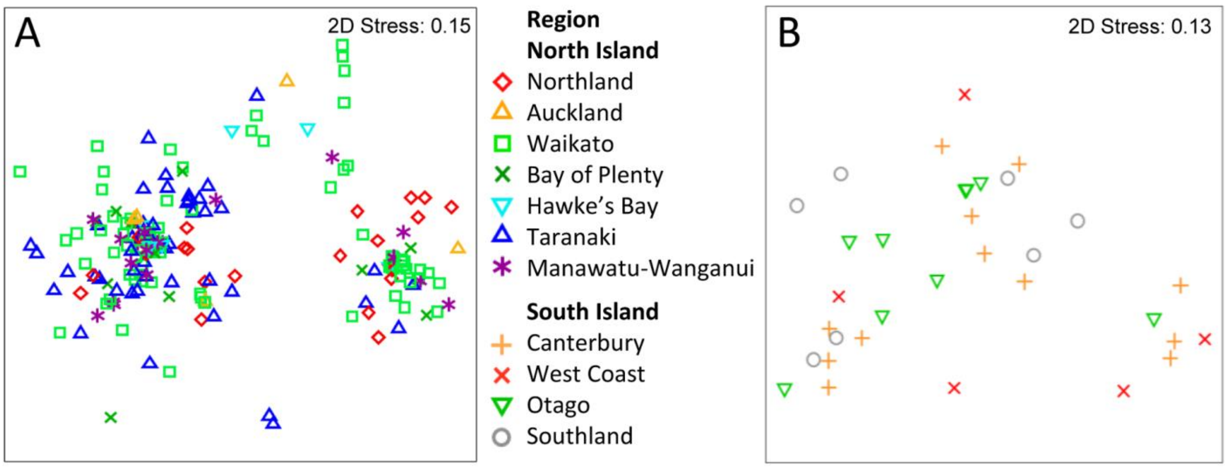

Technical Appendix Figure 3. Multidimensional scaling plots showing the genotypic clustering of bovine meat Shiga toxin–producing *Escherichia coli* O157:H7 isolates originating from the North Island (n = 200, 1 isolate excluded) and South Island (n = 33, 1 isolate excluded) of New Zealand. The plots were determined on the basis of the isolates' pulsed-field gel electrophoresis profiles. Clusters associated with regions are presented for isolates from the North Island (A) and the South Island (B).

	NZ Cattle	NZ Human	AU Cattle	AU Human	US Cattle	US Human
NZ Cattle (n = 40)		0.74 – 0.93	0.00 – 0.13	0.04 – 0.20	0.16 – 0.41	0.18 – 0.41
NZ Human (n = 363)	0.92		0.04 – 0.09	0.06 – 0.18	0.21 – 0.33	0.23 – 0.34
AU Cattle (n = 205)	0.06	0.06		0.57 – 0.79	0.27 – 0.43	0.05 – 0.13
AU Human (n = 79)	0.11	0.12	0.69		0.24 – 0.44	0.08 – 0.21
US Cattle (n = 143)	0.28	0.27	0.35	0.35		0.51 – 0.69
US Human (n = 179)	0.30	0.29	0.09	0.15	0.61	

Technical Appendix Figure 4. Proportional similarity index (PSI) values assessing the similarity of frequency distributions of Shiga toxin-encoding bacteriophage insertion (SBI) types among human and bovine Shiga toxin–producing *Escherichia coli* O157:H7 isolates originating from New Zealand (NZ), Australia (AU), and the United States (US). PSI values are presented in the lower half of the matrix with corresponding bootstrapped 95% confidence intervals in the upper half. Increased font size of PSI value shows higher degree of similarity.
